# Supplementary material for: Synapsin condensation controls synaptic vesicle sequestering and dynamics
Source: Nat Commun. 2023 Oct 23;14:6730. doi: 10.1038/s41467-023-42372-6 (PMC10593750; doi:10.1038/s41467-023-42372-6)
Supplement: Supplementary file 1 — Supplementary Information [file 41467_2023_42372_MOESM1_ESM.pdf]

Supplementary Information for:

Synapsin Condensation Controls Synaptic Vesicle Sequestering and Dynamics

Authors: Hoffmann Christian[1,*], Rentsch Jakob[2,*], Tsunoyama A. Taka[3,*], Chhabra Akshita[1], Aguilar Perez Gerard[1], Chowdhury Rajdeep[5], Trnka Franziska[1], Korobeinikov A. Aleksandr[1], Ali H. Shaib[5], Ganzella Marcelo[4], Giannone Gregory[6], Rizzoli O. Silvio[5], Kusumi Akihiro[3], Ewers Helge[2] & Milovanovic Dragomir[1*]

[1]Laboratory of Molecular Neuroscience, German Center for Neurodegenerative Diseases (DZNE), 10117 Berlin, Germany.
[2]Institute of Chemistry and Biochemistry, Freie Universität Berlin, 14195 Berlin, Germany.
[3]Membrane Cooperativity Unit, Okinawa Institute of Science and Technology Graduate University (OIST); Onna-son, Okinawa, 904-0495, Japan.
[4]Department of Neurobiology, Max Planck Institute for Multidisciplinary Sciences, 37077 Göttingen, Germany.
[5]University Medical Center Göttingen, Institute for Neuro- and Sensory Physiology, Germany; Biostructural Imaging of Neurodegeneration (BIN) Center, 37073 Göttingen, Germany; Excellence Cluster Multiscale Bioimaging, Göttingen, Germany.
[6]Interdisciplinary Institute for Neuroscience, University of Bordeaux, UMR 5297, F-33000 Bordeaux, France.

[*]These authors contributed equally to this work

✉Correspondence should be addressed to: dragomir.milovanovic@dzne.de

Supplementary Data contains:

- Supplementary Figures 1-14
- Supplementary Tables 1-2
- Supplementary Movies 1-3

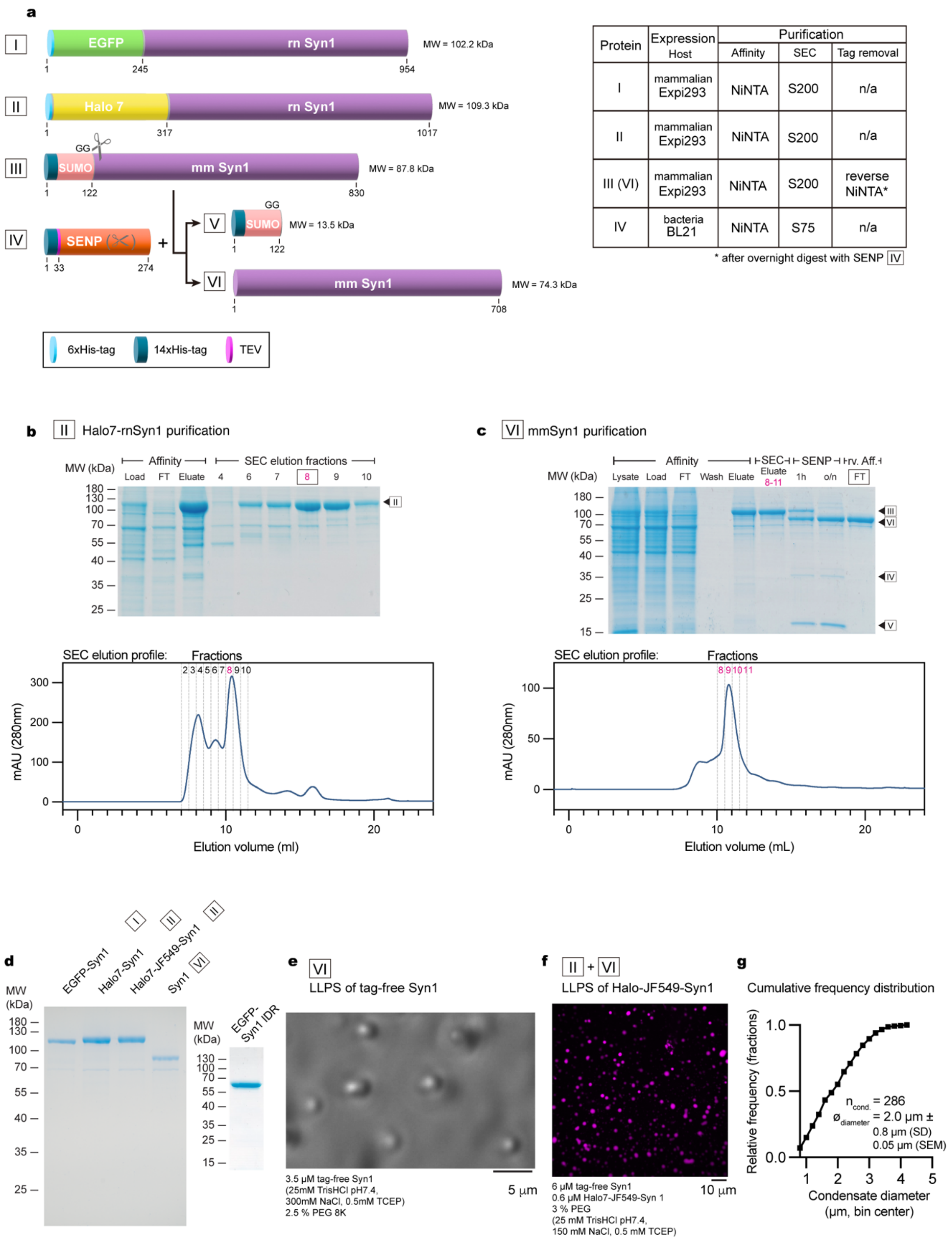

Supplementary Figure 1 | Recombinant proteins used for reconstitution of synapsin condensates. a, Left: Schemes of synapsin 1 proteins variants used for reconstitution. The cartoons are to scale. Right: Overview of the purification strategy for each construct. For details, see Material and Methods. **b,** SDS-PAGE gel for recombinant purified Halo7-synapsin 1. **c,** SDS-PAGE gel showing the purification of tag-free synapsin 1. **d,** SDS-PAGE

gel with all recombinant proteins used in this study (I: EGFP-synapsin 1; II: Halo7-synapsin 1; II*: Halo7*JF549-synapsin 1; VI: tag-free mmSynapsin 1). Right: EGFP-synapsin 1 IDR (amino acids: 416-705) **e,** Tag-free synapsin 1 forms condensates as visualized by phase contrast microscopy. **f,** JF549 labeled Halo7-synapsin 1 forms condensates. **g**, Cumulative size distribution of synapsin 1 condensates (mean 2 µm, standard deviation (SD) 0.8 µm, standard error of the mean (SEM) 0.05 µm).

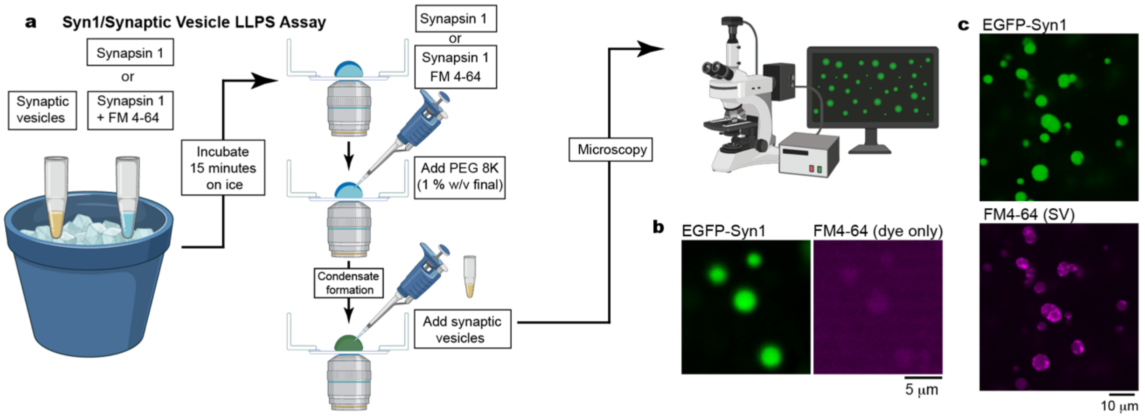

Supplementary Figure 2 | Reconstitution assay for visualizing SV/synapsin condensates. a, Scheme of the experimental pipeline. **b,** Exemplary fluorescence images of synapsin condensates with FM4-64 dye without SVs. **c**, Exemplary fluorescence images of synapsin 1/SV condensates.

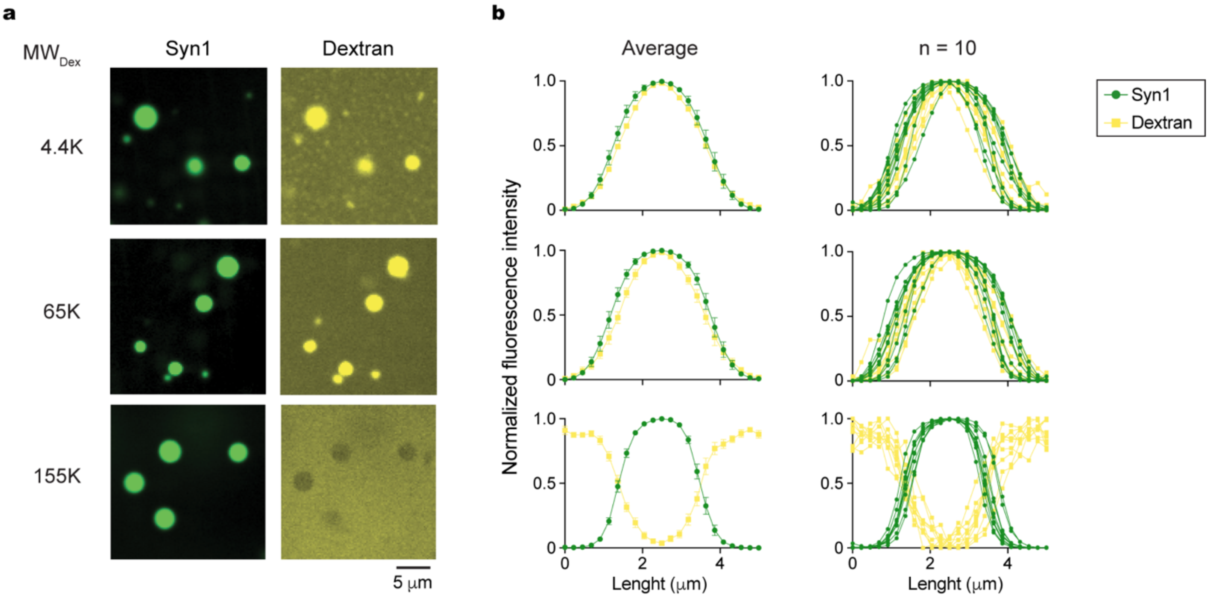

Supplementary Figure 3 | Synapsin condensates contain a meshwork that acts as a molecular sieve. a, Confocal images of EGFP-synapsin 1 condensates upon incubation with fluorescently-labeled dextran of 4.4 kDa (top), 65.85 kDa (middle), and 155 kDa (bottom) panels. Scale bar, 5 µm. **b**, Line profiles indicating either enrichment (for 4.4 kDa and 65.85 kDa) or exclusion (for 155 kDa) of fluorescently-labeled dextran. Left: an average line profile ± SEM; right: exemplary line profiles. For each condition ten condensates of similar size from three independent reconstitutions were analyzed.

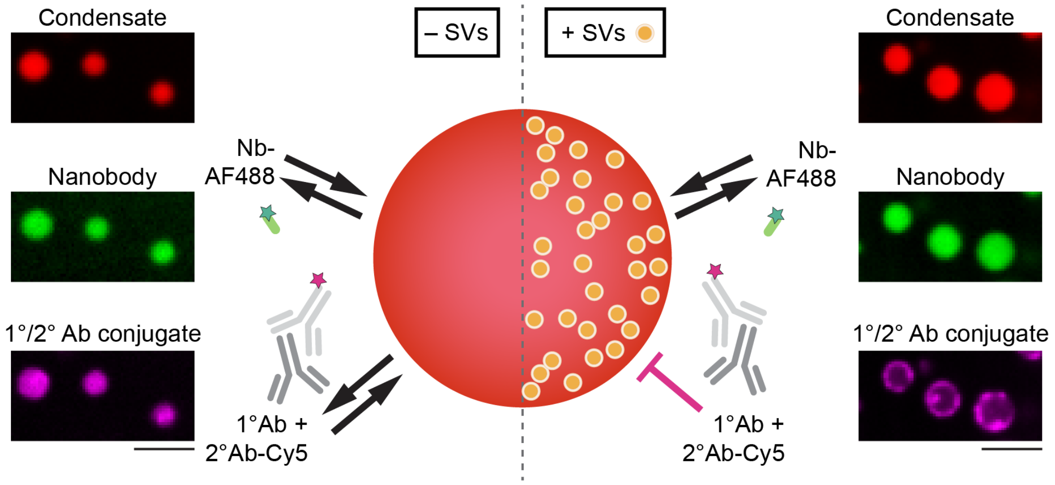

Supplementary Figure 4 | Differential partition of molecules into synapsin-drive condensates. Confocal images of Halo7*JF549-synapsin 1 condensates in the absence (left) or presence of synaptic vesicles (right). Condensates were co-incubated with a mixture of nanobody (Alexa Fluor 488-labeled nanobody against mouse IgG) and a primary/secondary antibody conjugate (primary: rabbit anti-LAMP1; secondary: Cy5-labeled goat-anti-rabbit). For each condition three independent reconstitutions were analyzed. Scale bars, 5 µm.

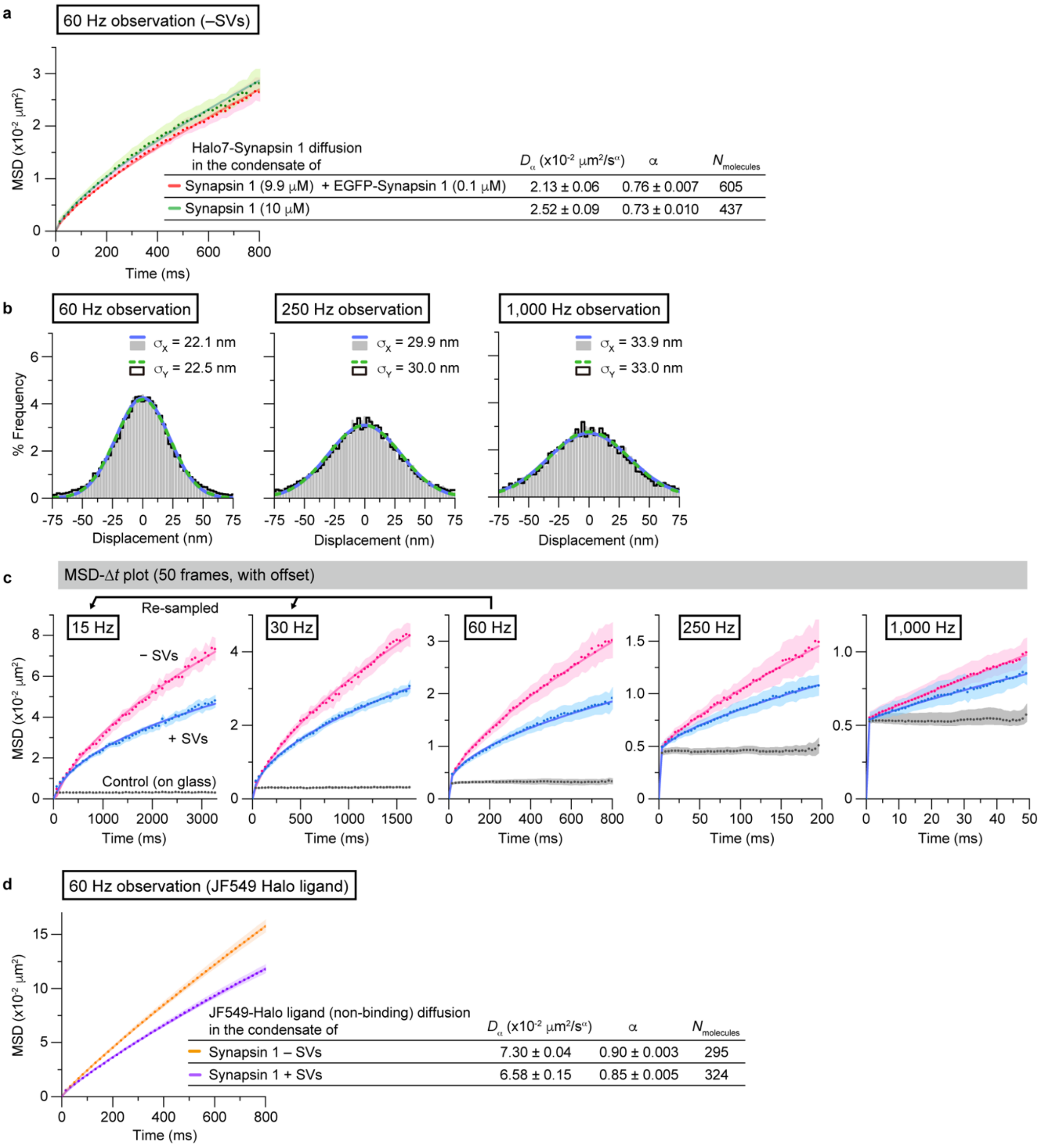

Supplementary Figure 5 | Control data for SMT employed for evaluating synapsin 1 diffusion in the SV/synapsin condensates. a, Halo7-synapsin 1 diffusion is not influenced by the presence of EGFP-synapsin 1 in the condensate (mean ± SEM). See Methods for details. **b,** Single-molecule localization precisions of JF549-labeled Halo7-synapsin 1 at three recording frame rates, determined for the molecules attached to the poly-L-lysine-coated glass-bottom dish. The standard deviations of the best-fit Gaussian functions provide the localization precisions. The number of observed spots were 23,800, 19,850, and 11,950 for frame rates of 60, 250, and 1,000 Hz, respectively. **c,** The MSD-Δ*t* plots for Halo7-synapsin 1 located in the inner volume of the condensate (mean ± SEM). In these plots, the offset values are not subtracted data. The plots after subtraction are shown in *Fig. 2e.* **d,** The MSD-Δ*t* plots for free JF549-Halo ligand molecules (not bound to Halo7 protein) located in the inner volume of the condensate (mean ± SEM). The diffusion is faster with less anomalies, compared with that of

the synapsin 1-bound dyes, but the effect of the SV inclusion in the synapsin 1 condensate is clearly visible; a decrease of the diffusion coefficient and an increase of the diffusion anomaly.

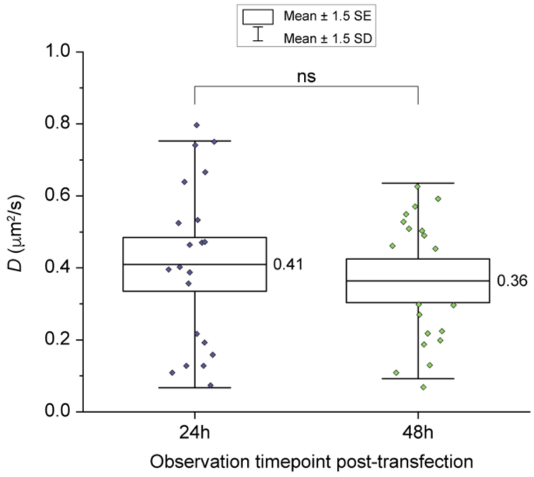

Supplementary Figure 6 | Confinement of expressed proteins in synaptic boutons is independent of the duration of expression. Mouse hippocampal neurons (14 days in culture) were transfected with mEOS3.2. Single-molecule tracking of mEOS3.2 was performed (100 Hz for 50 s) at either 24 h or 48 h post-transfection. Boxplot (mean ± 1.5 x SD (whiskers) and 1.5 x SE (box)) showing the geometric mean diffusion coefficients per tracking experiment (24 h, n = 21; 48 h, n = 20) of three independent neuronal preparations. Significance was tested using Mann-Whitney test (p = 0.71); ns = not significant.

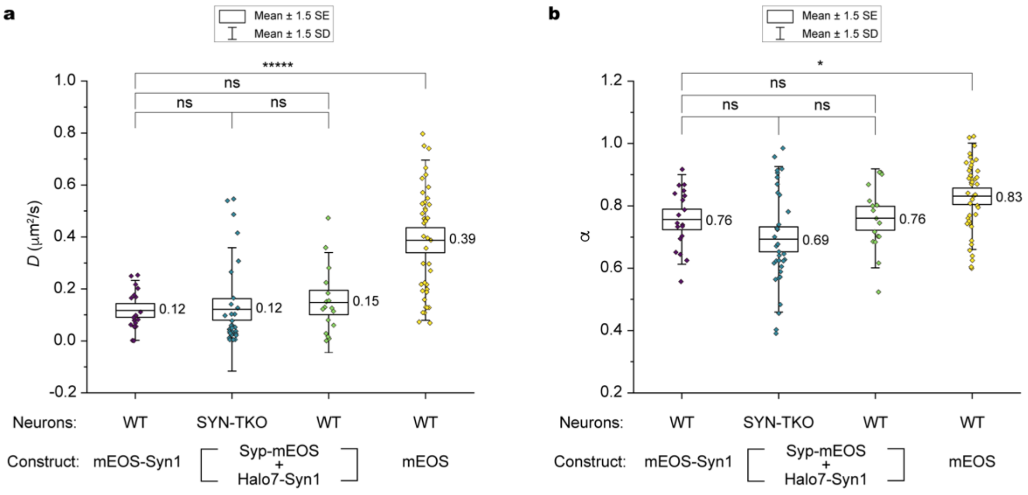

Supplementary Figure 7 | Synapsin confinement and diffusion rates are independent of synaptophysin/SVs. Single-molecule tracking experiments of synapsin 1 performed at 100 Hz for 50 s. (**a**) Boxplot (mean ± 1.5 x SD (whiskers) and 1.5 x SE (box)) showing the geometric mean diffusion coefficients per tracking experiment (WT mEos-Syn1, n = 19; SYN-TKO Syp-mEos and Halo7-Syn1, n = 33; WT Syp-mEos and Halo7-Syn1, n = 17, WT mEos, n = 41). Significance was tested using Mann-Whitney test (WT mEos-Syn1 vs WT mEos,

$p = 2.0\text{x}10^{-6}$; WT mEos-Syn1 vs WT Syp-mEos Halo7-Syn1, $p = 0.59$; WT mEos-Syn1 vs SYN-TKO Syp-mEos and Halo7-Syn1, $p = 0.12$; SYN-TKO Syp-mEos and Halo7-Syn1 vs WT Syp-mEos and Halo7-Syn, $p = 0.18$); asterisks indicate significance, ns = not significant. (**b**) Boxplot (mean ± 1.5 x SD (whiskers) and 1.5 x SE (box)) showing the mean coefficients of confinement (α) per tracking experiment (WT mEos-Syn1, n = 19; SYN-TKO Syp-mEos and Halo7-Syn1, n = 34; WT Syp-mEos and Halo7-Syn1, n = 17; WT mEos, n = 42). Significance was tested using Mann-Whitney test (WT mEos-Syn1 vs WT mEos, $p = 0.011$; WT mEos-Syn1 vs WT Syp-mEos Halo7-Syn1, $p = 0.95$; WT mEos-Syn1 vs SYN-TKO Syp-mEos Halo7-Syn1; $p = 0.083$; WT Syp-mEos and Halo7-Syn1 vs SYN-TKO Syp-mEos and Halo7-Syn1; $p = 0.084$); asterisks indicate significance, ns = not significant. Tracking data was acquired from at least three independent neuronal preparations. Mouse hippocampal neurons (14 days in culture) from wild-type (WT) or synapsin triple knockout (SynTKO) animals were transfected with mEOS3.2-synapsin 1. Rescue experiments were done by co-transfecting SynTKO neurons with synaptophysin-mEOS3.2 and Halo7-synapsin 1. Overexpression experiments were performed by co-transfecting wild-type neurons with Synaptophysin-mEOS3.2 and Halo7-synapsin 1. Additionally, neurons were transfected with soluble mEOS3.2 to assess the diffusion rate and confinement of a free diffusing protein in the axon. Of note, Halo7-synapsin 1 and mEOS3.2-synapsin 1 are significantly slower and more confined than soluble mEOS3.2 while not being affected by synaptophysin-mEOS3.2 expression. Thus, a molecular crowding alone is insufficient to account for the diffusion coefficients and coefficient of confinement of synapsin in synaptic boutons.

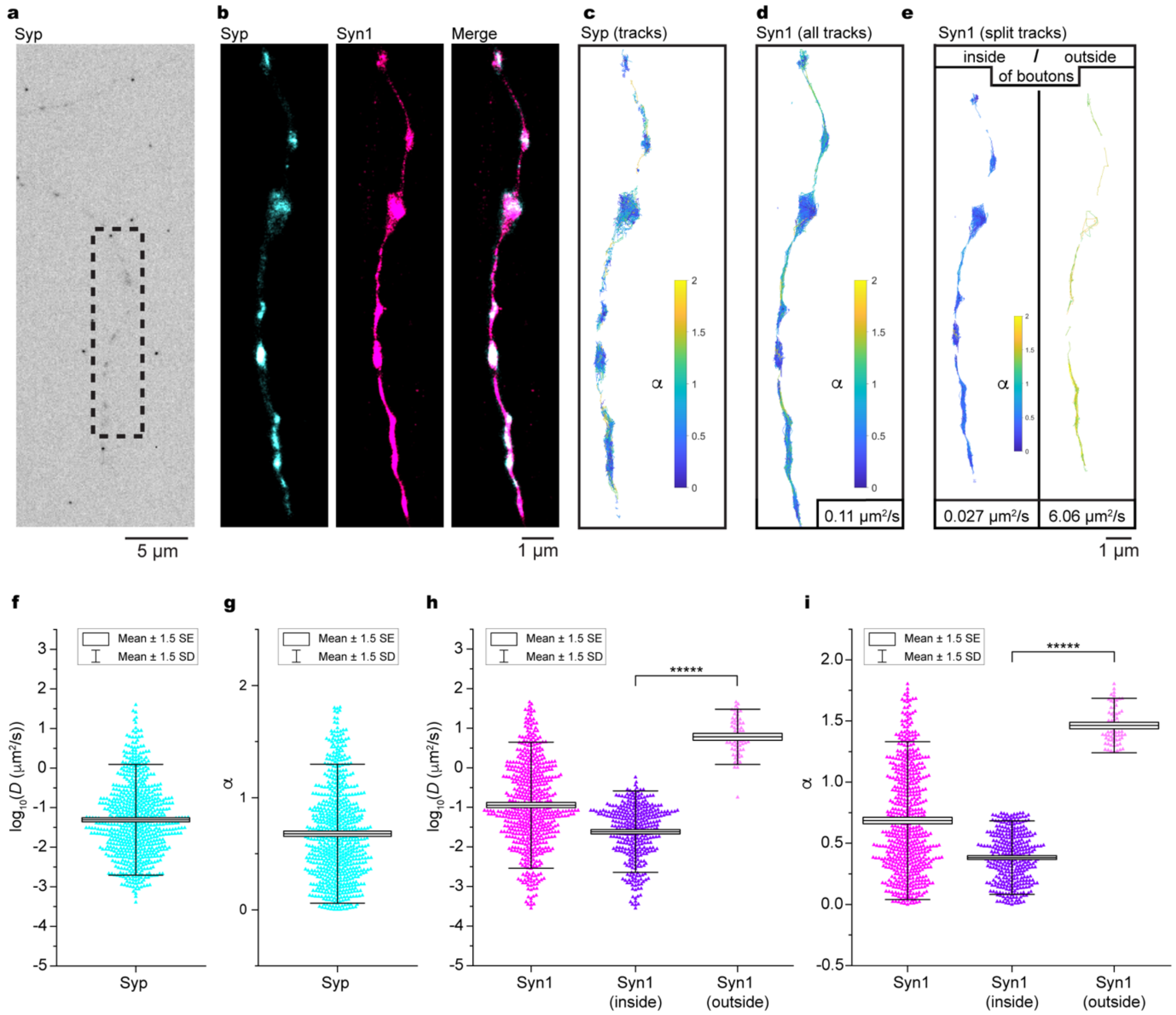

Supplementary Figure 8 | Synapsin 1 and synaptophysin are confined at synaptic boutons while maintaining their high motility. a, Widefield image of a neuron expressing synaptophysin-mEOS3.2. Mouse hippocampal neurons (14 days in culture) were transfected with Synaptophysin-mEOS3.2 (Syp) and Halo7-synapsin 1 (Syn1). **b**, Single-molecule localization reconstructions of proteins localized within dashed box in (*a*). Dual-color single-molecule tracking of Syp or Syn1 coupled to JF635 were performed at 100 Hz for 50 s. **c**, Map of all tracks (n = 690) of Syp within dashed box in (*a*) color-coded for coefficient of confinement (α). **d**, Map of Syn1 within dashed box in (*a*) color-coded for coefficient of confinement (α) (n = 596). **e**, Tracks of Syn1 filtered according to α (inside boutons (left, n = 355): 0.75> α > 0 or outside boutons (right, n = 68): 2 > α > 1.25. Values indicate the geometric mean diffusion coefficient for each panel. Boxplot (mean ± 1.5 x SD (whiskers) and 1.5 x SE (box)) showing diffusion coefficients (**f**) and α (**g**) for all tracks of Syp shown in (*c*). Boxplot (mean ± 1.5 x SD (whiskers) and 1.5 x SE (box)) showing diffusion coefficients (**h**) and α (**i**) for all tracks of Syn1 shown in (*d,e*). Significance was tested using Mann-Whtiney-Test; asterisks indicate significance; ns = not significant. Note that Syn1 is significantly slowed down (**h**, $p = 7.1\text{x}10^{-39}$) and confined (**i**, $p = 5.0\text{x}10^{-39}$) in synaptic boutons.

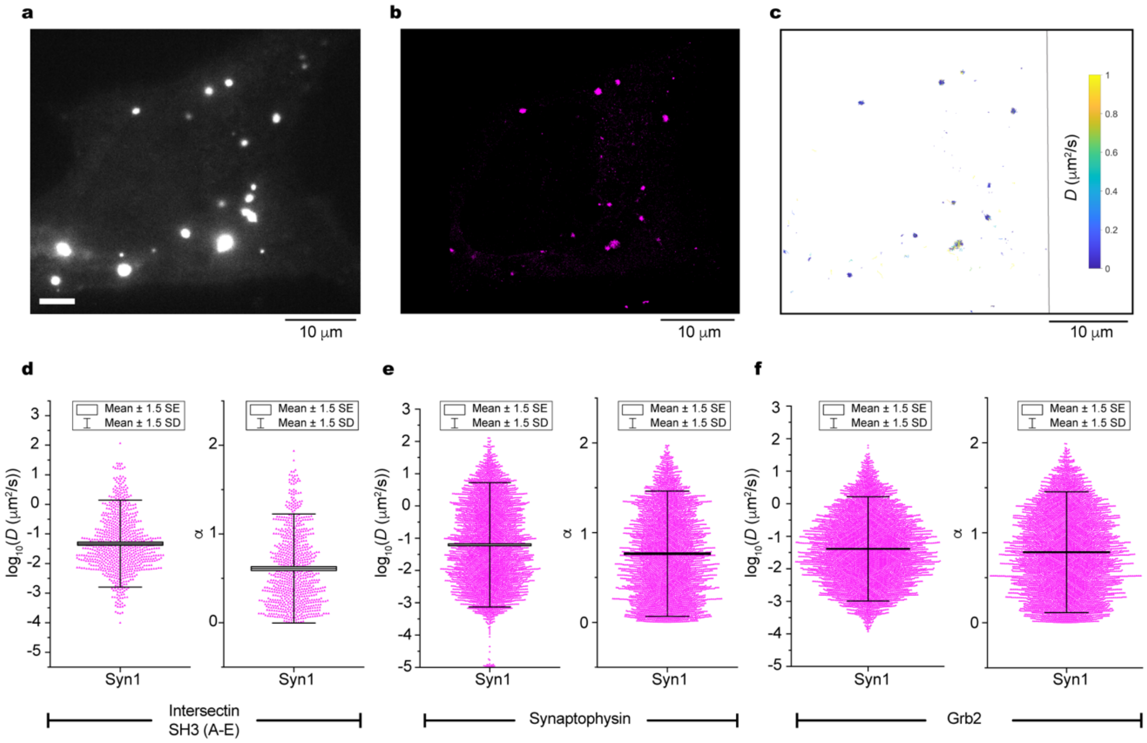

Supplementary Figure 9 | Synapsin 1-containing condensates reconstituted in non-neuronal cells recapitulate the motility pattern measured in synaptic boutons. CV-1 cells were transfected with EGFP-SH3(A-E) concatemer of Intersectin and mEOS3.2-synapsin 1 (Syn1). **a**, Widefield fluorescence image of synapsin 1-containing condensates in the cytosol. Single-molecule tracking measurements of Syn1 were performed at 100 Hz for 50 s. **b**, Reconstruction of proteins localized for cell in (*a*). **c**, Map of all tracks (n=768) for cell in (*a*) color-coded for diffusion coefficient. **d**, Boxplots (mean ± 1.5 x SD (whiskers) and 1.5 x SE (box)) showing diffusion coefficients (left) and the coefficient of confinement (α) (right) for all tracks of Syn1 shown in (c). **e**, the as in *d* but for the cells co-expressing Syn1 and untagged synaptophysin, **f**, the same as in *d* but for the cells co-expressing Syn1 and Grb2.

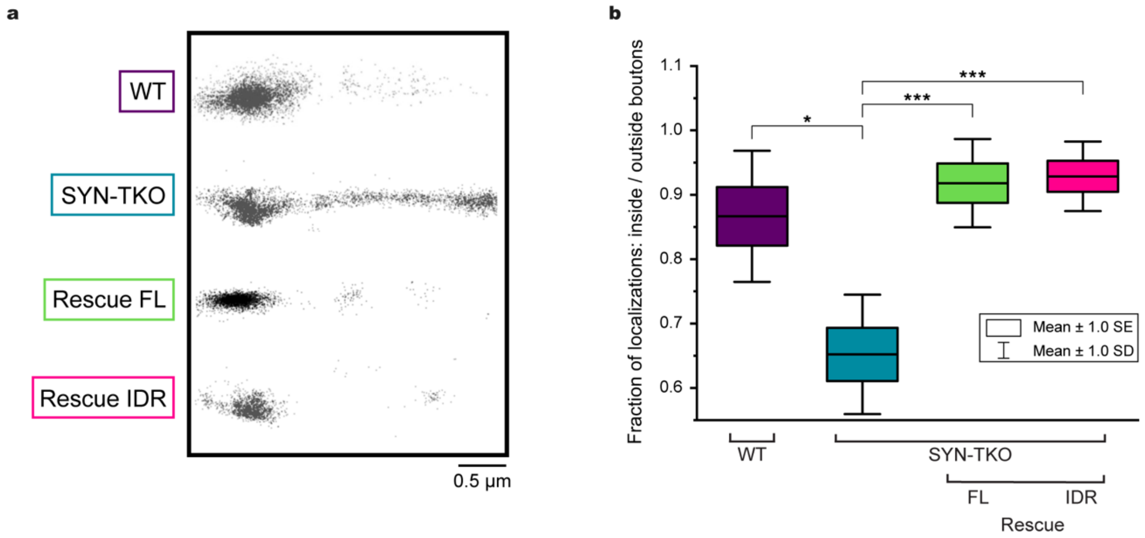

Supplementary Figure 10 | Quantification of the synaptic vesicle accumulation inside boutons. a, Representative single-molecule localization reconstructions of mouse hippocampal neurons (14 days *in vitro*) expressing synaptophysin-mEos3.2 (5000 frames, 10 ms exposure time) in neurons with different synapsin expressions: wild-type (WT), synapsin triple knock out neurons (SYN-TKO), SYN-TKO neurons rescued by expressing mCherry-synapsin 1 full-length (Rescue FL) or mCherry-synapsin 1 intrinsically disordered region amino acids 416-705 (Rescue IDR). Scale bar, 0.5 µm. **b**, Ratios of total number of single-molecule localizations of synaptophysin-mEos3.2 inside and up to 2 µm outside of boutons shown as a boxplot (mean ± 1.0 x SD (whiskers) and 1.0 x SE (box)) under the same synapsin expression patterns described in *a*. Significance was tested using unpaired *t-test* with equal variance assumed (WT vs SYN-TKO, $p = 8.3 \times 10^{-3}$; SYN-TKO vs Rescue-FL, $p = 8.8 \times 10^{-4}$; SYN-TKO vs Rescue-IDR; $p = 4.3 \times 10^{-4}$); n = 5 independent neuronal preparation for each expression pattern; asterisks indicate significance; ns = not significant.

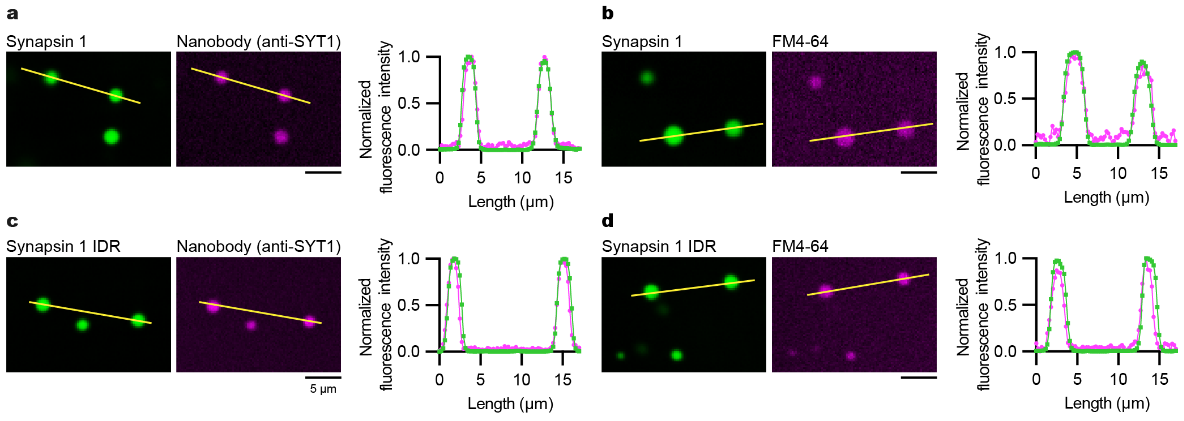

Supplementary Figure 11 | The full-length and the intrinsically disordered region of synapsin 1 sequester synaptic vesicles. a, Representative confocal images of the recombinant full-length synapsin 1 (10 µM) coincubated with synaptic vesicles (SVs, 3 nM) visualized using fluorescently-labeled Abberrior STAR 635P nanobody against synaptotagmin 1 (anti-SYT1). Left: Images, right: line profiles. **b,** The same as in *a* but SVs were visualized with lipophilic dye FM4-64. **c,** Representative confocal images of the recombinant intrinsically disordered region (IDR, amino acids 416-705) of synapsin 1 (10 µM) coincubated with synaptic vesicles (SVs, 3 nM) visualized using fluorescently-labeled Abberrior STAR 635P nanobody against synaptotagmin 1 (anti-SYT1). Left: Images, right: line profiles. **d,** The same as in *c* but SVs visualized with lipophilic dye FM4-64**.** Scale bars, 5 µm. All reconstitutions have been independently performed at least three times.

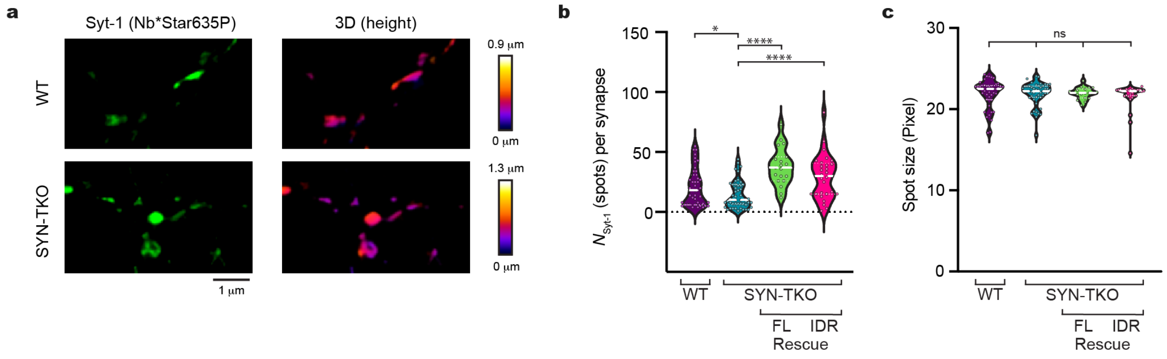

Supplementary Figure 12 | Quantification of the synaptic vesicle accumulation using 3D STED microscopy. a, Representative STED images of primary mouse hippocampal neurons stained with Aberrior STAR 635P-labeled nanobodies (Nb*Star635P) against synaptotagmin-1 (Syt-1), a *bona fide* synaptic vesicle protein. **b**, Quantification of number of Syt-1 positive spots in boutons of wild-type (WT; N = 41) neurons or synapsin triple knock-out (SYN-TKO; N = 48) neurons transfected with mCherry-synapsin 1 full-length (Rescue FL; N = 19) or mCherry-synapsin 1 intrinsically disordered region (IDR, amino acids 416-705; N = 28). **c**, Spot size distribution in all conditions analyzed in *b* indicating the consistent labeling pattern of Syt-1 nanobody. Significance was tested using unpaired, two-tailed *t-test*. Asterisks, significance; *, $p < 0.05$; ****, $p < 0.0001$; ns, not significant.

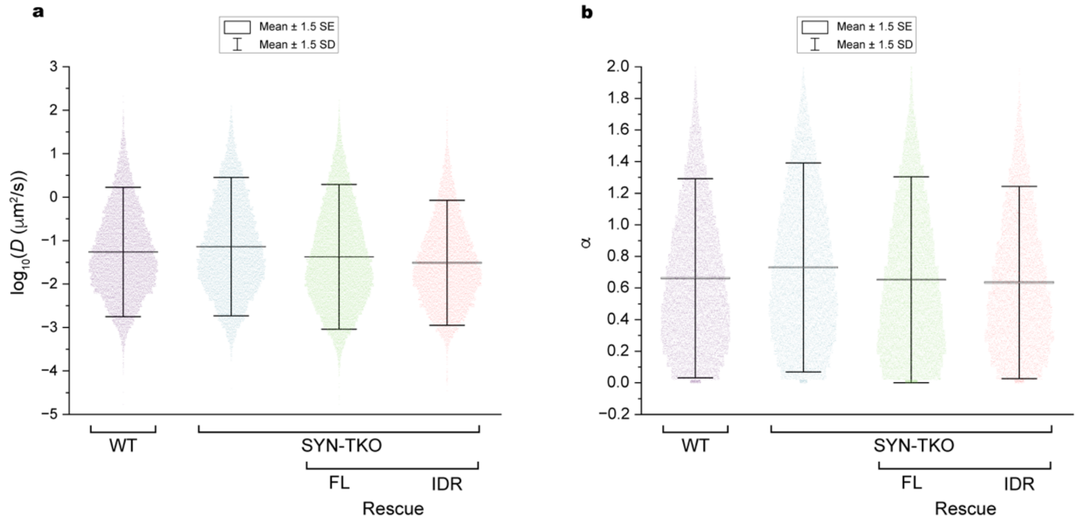

Supplementary Figure 13 | Analysis of all tracks confirms that synapsin determines the confinement and diffusion rates of synaptic vesicles (SVs). Single-molecule tracking experiments of synaptophysin-mEOS3.2 (Syp) performed at 100 Hz for 50 s. Mouse hippocampal neurons (14 days in culture) from wild-type or synapsin triple knockout (SYN-TKO) animals were transfected with Syp. Rescue experiments were done by co-transfecting SYN-TKO neurons with Syp and either full-length synapsin 1 (Rescue FL) or the intrinsically disordered region of synapsin 1 (Rescue IDR). Boxplots (mean ± 1.5 x SD (whiskers) and 1.5 x SE (box)) showing the diffusion coefficients (**a**) and the coefficient of confinement (α) (**b**) of all tracks (N = 77,675) of at least four independent neuronal preparations per condition.

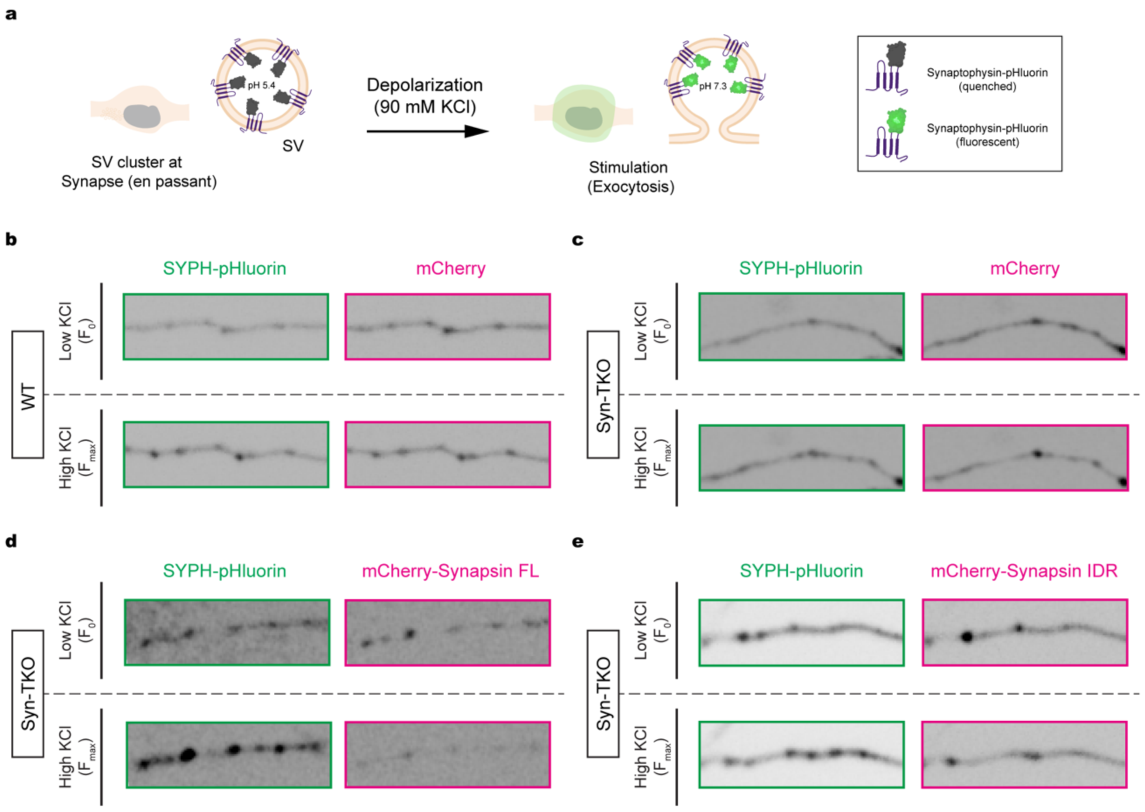

Supplementary Figure 14 | Functional assay for measuring SV release. a, Scheme of the assay. SV release measured as an increase in fluorescence of pH-sensitive EGFP variant tagged to the luminal region of synaptophysin (SYPH-pHluorin) upon chemical stimulation with KCl (90 mM). Representative images of wild-type neurons co-transfected with only a soluble red fluorophore (**b**) or SYN-TKO neurons co-transfected with a soluble red fluorophore (**c**), synapsin FL (**d**) or the synapsin IDR (**e**).

Supplementary Table 1. DNA sequences of (a) His14-SUMO_Eu1-mmSynapsin1a and (b) SYPH-pHluorin expression cassettes

a) …cmv-***Age*I**-His14-**SUMO_Eu1**-***Bgl*II**-mmSyn1a-*SV40*…
…cgccccattgacgcaaatgggcggtaggcgtgtacggtgggaggtctatataagcagagc<br>tGGTTTAGTGAACCGTCAGATCCGCTAGCGCT***ACCGGT***CGCCACCATGAGCAAGCATCACC<br>ATCATTCAGGCCATCACCATACCGGACACCACCATCATTCAGGCAGTCATCACCATTCCGG<br>A**TCTGCTGCGGGTGGCGAAGAAGATAAGAAACCGGCAGGTGGCGAAGGTGGCGGTGCCCAT**<br>**ATCAACCTGAAAGTGAAAGGTCAAGACGGCAACGAAGTCTTTTTCCGCATCAAACGTTCTA**<br>**CCCAGCTGAAAAAGCTGATGAACGCATACTGTGACCGTCAGTCTGTAGACATGAAGGCAAT**<br>**TGCTTTCCTCTTTAAGGGTCGTCGCCTACGTGCGGAAAGGACCCCGGATGAACTGGAAATG**<br>**GAAGATGGCGACGAAATCGACGCAATGCTGCACCAGACTGGAGGC*AGATCT***ATGAACTACC<br>TGCGGCGCCGCCTGTCGGACAGCAACTTCATGGCCAATCTGCCGAATGGGTACATGACAGA<br>CCTGCAGCGCCCGCAACCGCCCCCGCCGCCTCCCTCGGCCGCCAGCCCTGGGGCCACGCCC<br>GGCTCCGCGACAGCCTCTGCCGAGAGGGCCTCCACAGCTGCTCCAGTGGCTTCTCCAGCAG<br>CCCCTAGTCCTGGGTCCTCGGGGGGCGGCGGCTTCTTCTCGTCGCTGTCTAACGCGGTCAA<br>GCAAACCACAGCAGCCGCAGCCGCCACCTTCAGCGAGCAGGTGGGCGGTGGCTCTGGGGGC<br>GCAGGCCGCGGGGGCGCCGCCGCCAGGGTGCTGCTGGTCATCGACGAACCGCACACCGACT<br>GGGCAAAATACTTCAAAGGGAAGAAGATCCATGGAGAAATTGACATTAAAGTAGAGCAAGC<br>TGAATTCTCTGATCTCAATCTTGTGGCTCATGCCAATGGTGGATTCTCTGTGGACATGGAA<br>GTTCTTCGGAATGGAGTCAAAGTTGTGAGGTCTCTGAAGCCAGACTTTGTGCTGATCCGCC<br>AGCATGCCTTCAGCATGGCACGTAATGGAGACTACCGAAGTTTGGTCATTGGGCTGCAGTA<br>TGCTGGAATCCCCAGTGTAAACTCTTTGCATTCTGTCTACAACTTCTGTGACAAACCCTGG<br>GTGTTTGCCCAGATGGTTCGACTACACAAGAAGCTTGGAACAGAGGAATTCCCTCTGATTG<br>ATCAGACTTTCTATCCTAATCACAAAGAGATGCTCAGCAGCACAACATACCCTGTGGTTGT<br>GAAGATGGGCCACGCACATTCTGGGATGGGCAAGGTCAAGGTAGACAACCAACATGACTTC<br>CAGGATATTGCAAGTGTTGTGGCACTGACTAAGACATATGCCACTGCTGAGCCCTTCATTG<br>ATGCTAAATATGATGTGCGTGTCCAGAAGATTGGGCAGAACTACAAGGCCTACATGAGGAC<br>ATCAGTGTCGGGTAACTGGAAGACCAATACAGGTTCTGCTATGCTTGAGCAGATTGCCATG<br>TCTGACAGGTACAAGTTGTGGGTAGACACGTGCTCAGAGATTTTTGGGGGACTTGACATCT<br>GCGCAGTGGAAGCGCTGCATGGCAAGGACGGAAGGGATCACATTATTGAGGTGGTGGGCTC<br>CTCCATGCCACTCATTGGTGATCACCAGGATGAAGACAAGCAGCTCATCGTGGAACTTGTG<br>GTCAACAAGATGACTCAGGCTCTGCCTCGGCAGCCGCAGCGGGATGCTTCCCCTGGCAGGG<br>GCTCCCACAGCCAGTCTTCATCCCCAGGAGCCCTGACCTTGGGCCGCCAGACCTCCCAGCA<br>GCCTGCAGGTCCTCCTGCTCAACAACGACCCCCACCCCAGGGAGGCCCTCCACAGCCAGGC<br>CCAGGACCTCAGCGCCAGGGACCCCCGCTGCAGCAGCGCCCACCCCCACAAGGCCAGCAAC<br>ATCTTTCTGGCCTTGGACCGCCAGCTGGCAGCCCTCTGCCTCAGCGCCTACCAAGTCCCAC<br>CGCAGCACCTCAGCAGTCTGCCTCTCAGGCCACACCAGTGACCCAGGGTCAAGGCCGCCAG<br>TCGCGGCCAGTGGCAGGAGGCCCTGGAGCACCTCCAGCAGCGCGCCCACCAGCCTCCCCAT<br>CTCCACAGCGTCAGGCGGGGGCCCCGCAGGCTACCCGTCAGGCATCTATCTCTGGTCCAGC<br>TCCAACGAAGGCCTCAGGAGCCCCACCCGGAGGGCAGCAGCGCCAGGGCCCTCCCCAAAAA<br>CCCCCAGGCCCTGCTGGTCCCACTCGTCAGGCCAGTCAGGCAGGTCCCGGACCTCGCACTG<br>GGCCTCCCACCACACAGCAGCCCCGGCCCAGCGGCCCAGGTCCTGCTGGACGTCCCGCCAA<br>ACCACAGCTGGCCCAGAAACCCAGCCAGGATGTGCCACCACCCATCACCGCCGCTGCCGGG<br>GGACCCCCGCACCCCCAGCTCAACAAATCCCAGTCTCTGACCAATGCCTTCAACCTTCCAG<br>AGCCAGCCCCTCCCAGGCCCAGCCTTAGCCAGGACGAGGTGAAAGCTGAGACCATCCGCAG<br>CCTGAGGAAGTCTTTCGCCAGCCTCTTCTCCGACTGAGAGCTCAAGCTTCGAATTCTGCAG<br>TCGACGGTACCGCGGGCCCGGGATCCACCGGATCTAGATAACTGATCATAATCAGCCATAC<br>CACATTTGTAGAGGTTTTACTTGCTTTAAAAAACCTCCCACACCTCCCCCTGAACCTGAAA<br>CATAAAATGAATGCAATTGTTGTTGTT*AACTTGTTTATTGCAGCTTATAATGGTTACAAAT*<br>*AAAGCAATAGCATCACAAATTTCACA*…

b) …cmv-SYPH(M1-T190)-**pHluorin**-SYPH(C191-A240)-Linker-*SV40*…

…cgccccattgacgcaaatgggcggtaggcgtgtacggtgggaggtctatataagcagagctggtt
tagtgaaccgtcagatcCGCTAGCGCTACCGGACTCAGATCTCGAGCTCAAGCTTGCCACCATGGA
CGTGGTGAATCAGCTGGTGGCTGGGGGTCAGTTCCGGGTGGTCAAGGAGCCCCTTGGCTTC
GTGAAGGTGCTGCAGTGGGTCTTTGCCATCTTCGCCTTTGCTACGTGCGGCAGCTACACCG
GAGAGCTTCGGCTGAGCGTGGAGTGTGCCAACAAGACGGAGAGTGCCCTCAACATCGAAGT
CGAATTTGAGTACCCATTCAGGCTGCACCAAGTGTACTTTGATGCACCCTCCTGCGTTAAA
GGGGGCACTACCAAGATCTTCCTAGTTGGTGACTACTCCTCCTCGGCTGAATTCTTTGTCA
CCGTGGCTGTGTTTGCCTTCCTCTACTCCATGGGGGCCCTGGCCACCTACATCTTCCTGCA
GAACAAGTACCGAGAGAACAACAAAGGGCCAATGATGGACTTCCTGGCCACAGCAGTGTTC
GCTTTCATGTGGCTAGTTAGCTCATCCGCCTGGGCCAAAGGCCTGTCCGATGTGAAGATGG
CCACTGACCCAGAGAACATTATCAAGGAGATGCCTATGTGCCGCCAGACAGGAAACACA**GG**
CGGAAGTAAAGGAGAAGAACTTTTCACTGGAGTTGTCCCAATTCTTGTTGAATTAGATGGTGATGT
TAATGGGCACAAATTTTCTGTCAGTGGAGAGGGTGAAGGTGATGCAACATACGGAAAACTTACCCT
TAAATTTATTTGCACTACTGGAAAACTACCTGTTCCTTGGCCAACACTTGTCACTACTTTAACTTA
TGGTGTTCAATGCTTTTCAAGATACCCAGATCATATGAAACGGCATGACTTTTTCAAGAGTGCCAT
GCCCGAAGGTTATGTACAGGAAAGAACTATATTTTTCAAAGATGACGGGAACTACAAGACACGTGC
TGAAGTCAAGTTTGAAGGTGATACCCTTGTTAATAGAATCGAGTTAAAAGGTATTGATTTTAAAGA
AGATGGAAACATTCTTGGACACAAATTGGAATACAACTATAACGATCACCAGGTGTACATCATGGC
AGACAAACAAAAGAATGGAATCAAAGCTAACTTCAAAATTAGACACAACATTGAAGATGGAGGCGT
TCAACTAGCAGACCATTATCAACAAAATACTCCAATTGGCGATGGGCCCGTCCTTTTACCAGACAA
CCATTACCTGTTTACAACTTCTACTCTTTCGAAAGATCCCAACGAAAAGAGAGACCACATGGTCCT
TCTTGAGTTTGTAACAGCTGCTGGGATTACACATGGCATGGATGAACTATACAAAACCGGTCGCCA
GACAGGGAACACATGCAAGGAACTGAGGGACCCTGTGACTTCAGGACTCAACACCTCGGTGG
TGTTTGGCTTCCTGAACCTGGTGCTCTGGGTTGGCAACCTATGGTTCGTGTTCAAGGAGAC
AGGCTGGGCCGCCCCATTCATGCGCGCACCTCCAGGCGCCAGCGGCGGTTCCGGGGGTAGCGG
TGGCAGCTCCGGACTCAGATCTCGAGCTCAAGCTTCGAATTCTGCAGTCGACGGTACCGCGGGCCC
GGGATCCACCGGATCTAGATAA*AGCGGCCGCGACTCTAGATCATAATCAGCCATACCACATT*
TGTAGAGGTTTTACTTGCTTTAAAAAACCTCCCACACCTCCCCCTGAACCTGAAACATAAA
ATGAATGCAATTGTTGTTGTTAACTTGTTTATTGCAGCTTATAATGGTTACAAATAAAGCA
ATAGCATCACAAATTTCAC…

Supplementary Table 2. Primer sequences for plasmid construction

**#DML0331+0332** (***Age*I**, ***Bgl*II**)	**FW:** ATCA**ACCGGT**CGCCACCATGAGCAAGCATCACCATCATTCAGGCC<br>**RV:** ATCA**AGATCT**GCCTCCAGTCTGGTGCAG
**#DML0195+0196** (***Age*I**, ***Bgl*II**)	**FW:** ATCA**ACCGGT**GCCACCATGGCAGAAATCGGTACTGG<br>**RV:** TGAT**AGATCT**GCTGCCGCTGCCGGAAATCTCGAGCGTC
**#DML0438+0439** (***Nhe*I**, ***Age*I**)	**FW:** **CTAGC**GCCACCATGCATCACCATCACCATCAC**A**<br>**RV:** **CCGGT**GTGATGGTGATGGTGATGCATGGTGGC**G**
