# Supplementary material for: Synapsin condensation controls synaptic vesicle sequestering and dynamics
Source: Nat Commun. 2023 Oct 23;14:6730. doi: 10.1038/s41467-023-42372-6 (PMC10593750; doi:10.1038/s41467-023-42372-6)
Supplement: Supplementary file 3 — Description of additional supplementary files [file 41467_2023_42372_MOESM3_ESM.pdf]

Description of additional supplementary files

Supplementary Movie 1: Representative movies of for single molecule tracking of Halo7(JF549)-synapsin 1 molecules in condensates formed of 6 µM EGFPSynapsin 1 at 60 Hz (left), 250 Hz (middle), and 1,000 Hz (right). Scale bar, 5 µm.

Supplementary Movie 2: Twocolor single molecule tracking of primary hippocampal neurons expressing Halo7(JF535)-synapsin 1 and synaptophysin-mEOS3.2. Top, exemplary movies; bottom, reconstitution of protein localizations. Scale bar, 1 µm.

Supplementary Movie 3: Exemplary movies for pHluorin-based assay for SV release in neurons. Depolarization is triggered by high concentration (90 mM) of potassium chloride. Note the increase of fluorescence signal for synaptophysin-pHluorin and the dispersion of mSarletsynapsin 1 signal from synaptic boutons. Scale bar, 10 µm.
