# Supplementary material for: Synapsin condensation controls synaptic vesicle sequestering and dynamics
Source: Nat Commun. 2023 Oct 23;14:6730. doi: 10.1038/s41467-023-42372-6 (PMC10593750; doi:10.1038/s41467-023-42372-6)
Supplement: Supplementary file 7 — Reporting Summary [file 41467_2023_42372_MOESM7_ESM.pdf]

nature portfolio

Corresponding author(s): Dragomir Milovanovic

Last updated by author(s): September 21, 2023

Reporting Summary

Nature Portfolio wishes to improve the reproducibility of the work that we publish. This form provides structure for consistency and transparency in reporting. For further information on Nature Portfolio policies, see our Editorial Policies and the Editorial Policy Checklist.

Statistics

For all statistical analyses, confirm that the following items are present in the figure legend, table legend, main text, or Methods section.

n/a	Confirmed	
☐	☒	The exact sample size (*n*) for each experimental group/condition, given as a discrete number and unit of measurement
☐	☒	A statement on whether measurements were taken from distinct samples or whether the same sample was measured repeatedly
☐	☒	The statistical test(s) used AND whether they are one- or two-sided<br>*Only common tests should be described solely by name; describe more complex techniques in the Methods section.*
☒	☐	A description of all covariates tested
☒	☐	A description of any assumptions or corrections, such as tests of normality and adjustment for multiple comparisons
☐	☒	A full description of the statistical parameters including central tendency (e.g. means) or other basic estimates (e.g. regression coefficient) AND variation (e.g. standard deviation) or associated estimates of uncertainty (e.g. confidence intervals)
☐	☒	For null hypothesis testing, the test statistic (e.g. *F*, *t*, *r*) with confidence intervals, effect sizes, degrees of freedom and *P* value noted<br>*Give P values as exact values whenever suitable.*
☒	☐	For Bayesian analysis, information on the choice of priors and Markov chain Monte Carlo settings
☒	☐	For hierarchical and complex designs, identification of the appropriate level for tests and full reporting of outcomes
☒	☐	Estimates of effect sizes (e.g. Cohen's *d*, Pearson's *r*), indicating how they were calculated

Our web collection on statistics for biologists contains articles on many of the points above.

Software and code

Policy information about availability of computer code

Data collection	ImageJ, NIS-elements (Nikon)
Data analysis	ImageJ (1.54f, with Fiji package), MATLAB 2022b (9.13.0.2193358), Rstudio (2022.07.2+576, with R 4.2.2); Excel (2308); OriginPro 2021 (9.8.0.200); Graph Pad Prism (9.0), Illustrator (27.0)

For manuscripts utilizing custom algorithms or software that are central to the research but not yet described in published literature, software must be made available to editors and reviewers. We strongly encourage code deposition in a community repository (e.g. GitHub). See the Nature Portfolio guidelines for submitting code & software for further information.

Data

Policy information about availability of data

All manuscripts must include a data availability statement. This statement should provide the following information, where applicable:

- Accession codes, unique identifiers, or web links for publicly available datasets
- A description of any restrictions on data availability
- For clinical datasets or third party data, please ensure that the statement adheres to our policy

Source data are provided with this paper. All data generated or analyzed for this study are available within the paper and its associated Supplementary Information/ Source Data file.

Research involving human participants, their data, or biological material

Policy information about studies with human participants or human data. See also policy information about sex, gender (identity/presentation), and sexual orientation and race, ethnicity and racism.

Reporting on sex and gender	N/A
Reporting on race, ethnicity, or other socially relevant groupings	N/A
Population characteristics	N/A
Recruitment	N/A
Ethics oversight	N/A

Note that full information on the approval of the study protocol must also be provided in the manuscript.

Field-specific reporting

Please select the one below that is the best fit for your research. If you are not sure, read the appropriate sections before making your selection.

- [x] Life sciences
- [ ] Behavioural & social sciences
- [ ] Ecological, evolutionary & environmental sciences

For a reference copy of the document with all sections, see nature.com/documents/nr-reporting-summary-flat.pdf

Life sciences study design

All studies must disclose on these points even when the disclosure is negative.

Sample size	The sample sizes for all experiments were determined by the culture, observation field and ROI. No statistical methods were used to predetermine sample sizes, but sample sizes in this study are similar to those generally used in the field.
Data exclusions	No data were excluded from analysis.
Replication	All attempts at replication were successful. All results were independently replicated at least 3 times, as described in the figure legends.
Randomization	The study did not use group allocation. Mice were randomly chosen for making primary hippocampal cultures.
Blinding	For the in-vitro part including the reconstitution assays, blinding is not typically used. In fact, the proteins had to be separately purified with different protocols and the buffer conditions of different groups need to be carefully controlled by the experimentalists. The same is true for the reconstitution assays where the careful control of conditions is crucial for the experiments.<br>For the single-molecule tracking in cell line and primary hippocampal neurons as well as for the pHluorin-based assay to monitor exocytosis, we have done double-blind transfection by one experimentalist and the imaging by another experimentalist, to ensure the reproducibility. To ensure the reproducibility, we also repeated the single-molecule experiments in two different locations (Berlin and Bordeaux).

Reporting for specific materials, systems and methods

We require information from authors about some types of materials, experimental systems and methods used in many studies. Here, indicate whether each material, system or method listed is relevant to your study. If you are not sure if a list item applies to your research, read the appropriate section before selecting a response.

Materials & experimental systems

n/a	Involved in the study	
[ ]	[x]	Antibodies
[ ]	[x]	Eukaryotic cell lines
[x]	[ ]	Palaeontology and archaeology
[ ]	[x]	Animals and other organisms
[x]	[ ]	Clinical data
[x]	[ ]	Dual use research of concern
[x]	[ ]	Plants

Methods

n/a	Involved in the study	
[x]	[ ]	ChIP-seq
[x]	[ ]	Flow cytometry
[x]	[ ]	MRI-based neuroimaging

Antibodies

Antibodies used	FluoTag-X2*AF488 nanobody (NanoTag Biotechnologies, N1202-AF488); anti-LAMP1 antibody (Sigma, L1418), and goat-anti-rabbit

	IgG-Cy5 antibody (Cytiva, RPN999); FluoTag-X2 anti-Synaptotagmin 1 (NanoTag Biotechnologies, #N2302-Ab635P-L)
Validation	FluoTag-X2*AF488 has been validated by the vendor: https://nano-tag.com/product/fluotag-x2-anti-mouse-ig-kappa-light-chain/<br>anti-LAMP1 antibody has been validated by the vendor: https://www.sigmaaldrich.com/DE/en/product/sigma/l1418<br>IgG-Cy5 antibody has been validated by the vendor: https://www.sigmaaldrich.com/DE/en/product/sigma/gerpn999<br>FluoTag-X2 anti-Synaptotagmin 1: https://nano-tag.com/product/fluotag-x2-anti-synaptotagmin-1/

Eukaryotic cell lines

Policy information about cell lines and Sex and Gender in Research

Cell line source(s)	Wild-type C. aethiops kidney fibroblasts (CV-1) were a kind gift from the Helenius laboratory. CV-1 cells were grown in DMEM without phenol red, supplemented with 10% fetal bovine serum (FBS) and GlutaMAX (all Thermo- Fisher Scientific) at 37 °C in a CO_2-controlled humidified incubator.
Authentication	Authentication done by the source supplier.
Mycoplasma contamination	Cell lines tested negative for mycoplasma.
Commonly misidentified lines<br>(See ICLAC register)	Cell line used is not on the list.

Animals and other research organisms

Policy information about studies involving animals; ARRIVE guidelines recommended for reporting animal research, and Sex and Gender in Research

Laboratory animals	Wild-type mouse line: C57BL/6; Synapsin TKO mouse line: 129-Syn2tm1Pggd Syn3tm1Pggd Syn1tm1Pggd/Mmjax. Newborn p0/p1 pups were used for the preparation of primary hippocampal cultures.
Wild animals	The study did not involve wild animals.
Reporting on sex	Newborn pups of both sex were used for the preparation of neuronal cultures.
Field-collected samples	The study did not involve samples collected from the field.
Ethics oversight	All animal experiments were approved by the Institutional Animal Welfare Committees of the State of Berlin, Germany and Charité University Clinic (Berlin, DE), as well as State of Lower Saxony, Germany and Max Planck Institute for Multidisciplinary Sciences (Göttingen, DE).

Note that full information on the approval of the study protocol must also be provided in the manuscript.
